# Perceived barriers to the implementation of clinical pharmacy services in a metropolis in Northeast Brazil

**DOI:** 10.1371/journal.pone.0206115

**Published:** 2018-10-22

**Authors:** Genival Araujo dos Santos Júnior, Sheila Feitosa Ramos, André Mascarenhas Pereira, Aline Santana Dosea, Elton Matos Araújo, Thelma Onozato, Déborah Mônica Machado Pimentel, Divaldo Pereira de Lyra

**Affiliations:** 1 Laboratory of Teaching and Research in Social Pharmacy (LEPFS), Department of Pharmacy, Federal University of Sergipe, São Cristóvão, Sergipe, Brazil; 2 Department of Medicine, Federal University of Sergipe, Sergipe, Brazil; Robert Gordon University, UNITED KINGDOM

## Abstract

**Background:**

CLinical pharmacy services (CPS) are professional services provided by pharmacists, who use their skills and knowledge to take an active role in patient health. These services have expanded in health systems around the world. However, it is important to have a comprehensive understanding of factors that may hinder the implementation of CPS in health systems.

**Objective:**

To identify pharmacists’ and managers’ perceptions of barriers regarding the implementation of CPS in some public health units in a metropolis in Northeast Brazil.

**Methods:**

This is a qualitative study based on focus groups and semi-structured, face-to-face, in-depth interviews. Participants were health-system pharmacists and managers, selected based on their direct participation in the implementation process. Focus groups were carried out with the pharmacists, and interviews were carried out with managers. The audio and videos were transcribed verbatim in full, and were independently analyzed using content analysis. This study was approved by the Brazilian Committee of Ethics in Research and all participants signed informed consent forms.

**Findings:**

There were two focus groups and five interviews. The discussions generated 240 minutes of recordings. The health-system pharmacists and managers expressed barriers were allocated into five categories to facilitate a comprehensive understanding of the implementation of CPS; these barriers were related to: the local healthcare networks, the healthcare team, the pharmacists, the implementation process, and the patients.

**Conclusions:**

This study revealed the perceptions of barriers associated with the participants involved in the implementation of CPS in some public health units in a metropolis in Northeast Brazil. The barriers reflect the challenges to be overcome in the CPS implementation process in the health systems.

## Introduction

Clinical Pharmacy Services (CPS) can be defined as professional services provided by pharmacists, who use their skills and knowledge to take an active role in patient health, through effective interaction with both patients and other healthcare professionals [1]. In these services, pharmacists have started to develop an important role in patient care process, reducing medication errors [2,3], reducing costs of drug therapy [4,5], and improving patient health conditions [6,7].

In this way, the CPS have expanded in different settings into the health systems [8–10]. In Brazil it would not be different, because CPS have been implemented in the Brazilian Health System (SUS) in the last ten years [11,12]. It is estimated that there are more than 2,500 pharmacists developing CPS [13], many of whom work in health units at SUS [14]. This expansion can be explained by measures taken by the Brazilian Federal Pharmacy Council, which has regulated the clinical activities of the pharmacists [15], and by Federal Government initiatives, such as the National Qualification Program for Pharmaceutical Service, which has implemented CPS in the SUS [16].

However, despite the advancement of these services in health systems around the world, the CPS implementation process is challenging, complex, and influenced by multiple factors [17,18]. Thus, it’s important to have a comprehensive understanding of factors that may hinder the implementation of CPS in health systems Additionally, there is a gap in studies that discuss the barriers to CPS implementation in SUS.

This study aimed to identify the perceptions of barriers of pharmacists and managers who participated in the CPS implementation process developed by the Brazilian Ministry of Health in some public health units in a metropolis in Northeast Brazil.

## Methods

### Study design

This was a qualitative study involving focus groups and interviews, in order to capture a comprehensive understanding of the implementation of CPS in some public health units in a city in northeast Brazil. The focus groups and interviews were conducted in April and August 2016, respectively. This study used the recommendations proposed by consolidated criteria for reporting qualitative research [19].

### Study context

The present study investigated the CPS implementation process was carried out by the Brazilian Ministry of Health, from July 2015 to March 2016. First, a collaborative partnership was agreed between the Brazilian Ministry of Health and the local health authority to ensure the provision of minimum structural resources for the implementation of CPS. Second, an expert team with substantial previous experience was hired to implement CPS. This expert team was part of the Laboratory of Teaching and Research in Social Pharmacy of the Federal University of Sergipe. In the last step, CPS were implemented through the theoretical and practical training based on patient care process [20] of 42 health-system pharmacists.

The pharmacists should comply with all stages of the implementation process and achieve a performance above 70% in the criteria for accreditation. The accreditation criteria proposed by the Brazilian Ministry of Health were divided into two axes: a) theoretical: directed study, and theoretical evaluation; b) practical: seminars to discuss real clinical cases, attendance of at least 30 first-time patients and 25 return visits, and performance in the patient care process. Therefore, at the end of the implementation process, 23 pharmacists were accredited by the Brazilian Ministry of Health. The whole process of implementation and pharmacists’ accreditation are described in detail in previous studies [21].

### Setting

This study was performed in Recife city, the ninth-largest metropolitan area in Brazil, with 3,940,456 residents. In the local healthcare network, the CPS were implemented in varied settings in the Brazilian health system, such as primary and secondary health units, mental health centers, emergency rooms, and drug distribution centers (settings that supply pharmacies with drugs and devices). In these workplaces, there were no CPS with systematized and documented pharmacists’ work processes before the CPS implementation. The pharmacists focused on logistics activities. Ethics approval was granted by the Brazilian committee of ethics in research (CAAE 35440114.0.0000.0008).

### Participants

Participants were health-system pharmacists and managers who were selected based on their direct participation in the implementation process. The health-system pharmacists were: (i) accredited pharmacists, who started the CPS implementation process, fulfilled all stages of the implementation process and implemented CPS at their workplaces; and (ii) non-accredited pharmacists, who started the CPS implementation process, but they gave up the implementation process and did not implement CPS at their workplaces. The managers were the five managers involved in CPS implementation, such as director and coordinators.

### Focus groups and interviews structure and moderation

The health-system pharmacists were allocated in two focus groups: accredited and non-accredited pharmacists. We could not make focus group with managers, because they had different hierarchical levels among them and it was impossible to gather them in the same session. Thus, semi-structured, face-to-face, in-depth interviews were conducted with five managers.

Focus groups and interviews were performed according to recommendations in the literature [19] and were moderated by researchers (ASD and GASJ). The questions were formulated from a brainstorming meeting with the authors; these questions asked about each participant's perceptions of barriers, facilitator and strategies to implement CPS. Prior to the focus group and interview sessions, participants signed the informed consent form authorizing the researchers to use the data collected.

### Data collection and analysis

The moderators maintained a neutral relationship with study participants and created a stimulating environment for the exchange of views. Additionally, they asked open-ended questions and only asked a new question or stop collecting information when new data tended to be redundant of the data already collected (data saturation). The focus group and interviews were recorded on video and/or voice.

The data collected were transcribed verbatim in full by three researchers (GASJ, AMP, and SRF). They immersed themselves in the data through multiple readings, and all transcribed data were independently analyzed using the Bardin content analysis technique [22].

This technique is composed of three phases: (1) pre-analysis: the material to be analyzed is organized, the initial ideas are systematized, and text cuttings are made in document analysis. The analysis was done by three researchers (GASJ, AMP, and SRF) independently, with a coding system; (2) the exploitation of the material: the data are aggregated into themes. (3) the interpretation: the material is interpreted, and inferences are made, in an inductive way, through consensus meetings that were held among the research team members.

### Rigour and trustworthiness

The focus groups and interviews sessions were moderated by experienced researchers. They used the data saturation like models of saturation. In addition, the data analysis was performed by team members with varied backgrounds, one of them (GASJ) was involved in the data collection, and two of them were external researchers (AMP and SRF). Finally, all analyzed data were carefully reviewed by two senior researchers, one of them (DPLJ) was involved in the CPS implementation process, and the one of them (DMMP) was an external research with expertise in qualitative research. These measures were taken to maintain trustworthiness of the research findings.

## Results

The focus groups with accredited pharmacists and non-accredited pharmacists were formed by eight and five pharmacists, respectively. No manager refused to participate in the interviews. The discussions generated a 104-minute recording with accredited pharmacists and a 36-minute recording with non-accredited pharmacists. The interviews had a duration of 100 minutes (average per interviewee: 20 minutes).

The participants expressed several barriers to the implementation of CPS. The barriers were allocated into five categories to facilitate comprehensive understanding of the implementation; these barriers were related to: the local healthcare networks, the healthcare team, the pharmacists, the implementation process, and the patients. Table 1 shows the barriers and the categories that emerged from the focus groups and interviews. The facilitator and strategies to implement CPS are described in a previous study [23].

**Table 1:** Perceived barriers to the implementation of clinical pharmacy services in some public health units in a metropolis in Northeast Brazil.

Categories	Accredited pharmacists	Non-accredited pharmacists	Managers
Local healthcare network	- Health professionals’ stoppages and strikes. - Shortage of drugs and devices. - Lack of adequate physical structure in some health units. - Unawareness of some managers regarding CPS.	- Shortage of drugs and devices. -Lack of adequate physical structure in health units. - Dismissals and lack of sufficient human resources. - Physical distance between some health units and the pharmacists' workplaces.	- Unfavorable political, administrative, and economic environment. - Shortage of drugs and devices. - Lack of adequate physical structure in some health units. - Lack of information about rational drug use in the local healthcare networks. -Managers’ resistance to implement the CPS.
Healthcare team	- Unawareness of the healthcare team about the pharmacists’ work.	- Healthcare team resistance to implement CPS.	- Lack of understanding of the healthcare team about the implementation of CPS. - Unawareness of the healthcare team about the pharmacists’ work.
Pharmacists	- Insufficient clinical education and training during undergraduate degree in Pharmacy. - Difficulty in recruiting patients. - Difficulty understanding the implementation of CPS. - Lack of adaptation among the healthcare team.	- Difficulty in reconciling the clinical and logistic activities. - Gaps in pharmacist-health unit communication. - Decline of the pharmacist and healthcare team relationship. - Pharmacists’ resistance to implement the CPS. - Difficulty in recruiting patients.	- Insufficient clinical education and training during undergraduate degree in Pharmacy. - Difficulty in reconciling the clinical and logistic activities. - Pharmacists who graduated a long time ago. - Lack of initiative and proactivity in the healthcare team. - Pharmacists resistance to implement the CPS.
Implementation process of the CPS	- Inappropriate period to implement CPS. - Short period to implement CPS. - CPS not tailored to the health unit and patients. - Poor marketing strategies. - Lack of prior evaluation of pharmacists’ clinical competences.	- Inappropriate period to implement the CPS.	- Lack of electronic health records (documentation system). - Need for adequate physical structure in the health units, and a change in the pharmacists' work processes.
Patients	-Lack of understanding about CPS.	- Resistance and lack of awareness among the patients regarding CPS.	Not related.

### Barriers related to the local healthcare networks

The participants mentioned problems related to physical and human resources. They were unanimous in pointing the physical structure limitations in some health units (including lack of physical areas and material resources, such as furniture, internet, computer equipment, and devices) and a lack of a private area for pharmacists’ clinical activities. Additionally, the accredited pharmacists reported stoppages and strikes among the healthcare professionals (physicians, nurses, pharmacists, pharmacy staff, community health workers, administrative staff), and non-accredited pharmacists reported dismissals and lack of sufficient human resources (pharmacy and administrative staff).

The pharmacists and managers identified that there were problems related to drug logistics management and planning. A shortage of drugs and devices may have directly influenced the pharmacists’ activities. The pharmacists reported that the patients were dissatisfied and discredited with their work. In contrast, the managers considered that the structural problems and shortage of drugs and devices were related to the specific and transitory stage of City.

*“There was a lack of many drugs and devices […] This really affects the pharmacists' work*, *because we are working with rational drug use*, *but there are no drugs”* (accredited pharmacist G).

The pharmacists discussed issues related to the health service delivery profile of some health units (mental health centers, emergency rooms, and drug distribution centers). According to them, some workplaces did not have a favorable care profile for straightforward implementation of CPS.

*“In the emergency room*, *we can’t easily recruit the patients*. *They stay for a little time in there*. *Soon after they are transferred to other services or are discharged”* (accredited pharmacist E).

*"In the mental health center*, *not all patients can have access to the CPS*. *For some patients*, *I scheduled a meeting and they did not come because of their clinical profile*. *They simply forget*. *We cannot contact them because they do not answer the phone*. *Their relatives are sometimes not involved in the patient's life"* (non-accredited pharmacist C).

The managers also declared that the lack of information about rational drug use in the local healthcare networks might have contributed to difficulty in the planning of strategies to facilitate the implementation of CPS.

*“We can only have drug management data, we cannot have the data about evidences of the clinical impact of the pharmacists in the patients”* (manager A).

### Barriers related to the healthcare team

The pharmacists and managers reported barriers related to the interaction between pharmacists and healthcare team.

*“What I find it very difficult is the lack of understanding of some healthcare professionals about the pharmacists’ clinical activities*” (accredited pharmacist F).

### Barriers related to the pharmacists

Accredited pharmacists stated that an important barrier was insufficient clinical education and training during undergraduate degree in Pharmacy. Managers endorsed these concerns.

*"In general, the pharmacists do not have a training to take care of patients, at the University. They finish their degree in pharmacy and are not ready to deal with the patients" (manager C)*.

Other related barriers were the difficulty in reconciling clinical and logistic activities, difficulty in recruiting patients to CPS, and barriers related to poor interaction with healthcare professionals, such as communication failures and lack of initiative and proactivity to work as part of the healthcare team.

*“I was very overloaded with tasks*. *I often had to complete the administrative tasks*. *Consequently*, *I had no time to perform clinical activities”* (non-accredited pharmacist E).

*"I had problems on understanding how the CPS would actually be in practice*. *Consequently*, *I had difficulty communicating to the healthcare team how I would take care of the patients”* (accredited pharmacist B).

### Barriers related to the implementation process

Accredited and non-accredited pharmacists highlighted the poor choice of implementation period, which coincided with long holidays (summer vacation, Christmas and new year period, carnival, etc.) and vacations for most of the healthcare team. Additionally, accredited pharmacists mentioned the short period given to implement CPS (nine months), which may have compromised its implementation. These barriers can also be considered as failures of the CPS implementation process.

*"The time was very short [*…*] This construction of knowledge needs a maturity time [*…*] It is like the story of the butterfly's flight*. *The butterfly can only fly when its wings are ready to fly*. *There is no use in opening the cocoon before its maturity time*” (accredited pharmacist H).

The managers indicated that the lack of electronic health records to store patients' health information collected by pharmacists was another barrier, because these health records should be shared across different health care settings and healthcare professionals. Additionally, accredited pharmacists discussed that a lack of prior evaluation of the pharmacists’ clinical competencies, and poor marketing strategies, prevented a rapid implementation of CPS.

### Barriers related to the patients

Pharmacists were the only participants to report patient-related barriers. The managers did not discuss this issue.

*“One of the greatest difficulties was the lack of knowledge of both the population and the healthcare professionals about the pharmacist's clinical activities”* (Accredited pharmacist E).

## Discussion

### Barriers related to the local healthcare networks

The lack of adequate physical structures in the health units was a barrier to the implementation of CPS. Structural problems can impact on the quality of care provided, the privacy required to perform CPS, and the construction of a therapeutic relationship between the pharmacist and their patients [11,24,25]. In Brazil, there is legislation that regulates the provision of adequate physical structures. Nevertheless, we found discrepancies among the official documents, the reports of the participants and other studies [26–28]. Therefore, it is crucial that health decision-makers keep abreast of planning, programming, drafting, evaluation, and monitoring of healthcare facilities.

Other mentioned barrier was the insufficient human resources. Studies have found similar findings regarding the insufficient workforce [25,29–31]. Brazinha and Fernandez-Llimós [32] showed that an insufficient pharmacy workforce impacted on the high pharmacist workload in terms of logistic and administrative tasks, and lead to a reduced focus on clinical activities. Therefore, health systems must ensure sufficient human resources to collaborate with pharmacists on bureaucratic, administrative, and clinical tasks.

In our study, the reported lack of medications and inputs directly influenced all healthcare networks. Heiskanen et al. [33] suggests that drug shortages may cause patient dissatisfaction and increase the workload problems of pharmacy staff. Moreover, in Brazil we have a peculiar situation that the society goes through which is the phenomenon of medicalization of life, an erroneous belief about that the medicine could solve all or the great majority of the health problems [34,35]. In this situation, the drugs assume a fundamental role in the health care process [36,37]. Thus, we infer that the Brazilian situation turned the shortage of drugs into a barrier, because it caused dissatisfaction and discredit on the patients with the pharmacist's clinical role.

The health service delivery profile of some health units was mentioned as a barrier. Although emergency rooms and mental health care centers are challenging workplaces, studies show that pharmacists perform clinically significant interventions, optimize drug use, reduce medication errors, and decrease drug-related problems [38,39]. Thus, the Thus, the literature endorses the core elements of patient care process, which asserts that any patients using prescription and non-prescription medications, herbal products, and dietary supplements could potentially benefit from CPS [20].

Lastly, a barrier reported by the pharmacists was a lack of information about rational drug use in the local healthcare networks; this has also been reported in other studies [25,40]. This barrier may have led to managers’ resistance to implement CPS. In fact, there are few studies investigating drug utilization in this municipality [41–43], and there are few national studies that evaluate drug utilization. The first one was National Survey on Access, Use and Promotion of Rational Use of drugs in Brazil [44]. However, the results of the survey only began to be published in 2016, after the process of implementation of CPS.

### Barriers related to the healthcare team

A collaborative relationship between pharmacists and healthcare professionals, with the aim of improving patient and health system outcomes, has become an important goal to be achieved since pharmacists changed their original focus from drug supply towards a focus on patient care [45–47]. Studies indicate that barriers related to pharmacist-healthcare team interaction may hinder the CPS implementation process [32,48]. Accordingly, inter-professional collaborative relationships should be encouraged to help decrease resistance and increase understanding and awareness among the healthcare team regarding the implementation of CPS.

### Barriers related to the pharmacists

The insufficient clinical education and training during undergraduate degree in Pharmacy was a barrier that can be explained by the historical difficulties that colleges and schools of pharmacy have in adapting to the new role of pharmacists in patient care process, particularly in emerging countries such as India [49], Jordan [25], Sudan [50], China [31], and Brazil [26,51]. In contrast, in the United States of America (USA), where CPS first started, some authors describe events in pharmaceutical education, training, practice, and research that have occurred over the years [52,53]. This history in the USA shows that CPS have a promising future, but that it is necessary to promote advances in education and clinical pharmacy research so that pharmacists achieve a high level of knowledge, skills, and attitudes in patient care process.

Pharmacists and managers related to the difficulty in reconciling clinical and logistic activities, which is consistent with the findings of some studies [30,32]. In the present study, this barrier is related to the pharmacists' workload, the lack of sufficient human resources and the poor delegation of pharmacy tasks among members of the pharmacy staff. The lack of time, lack of staff, and large workload are challenges experienced by pharmacists when trying to perform clinical activities [54,55]. Concerning this issue, the literature suggests that pharmacy technicians can assist pharmacists in logistic activities, freeing up pharmacists to devote their attention to other areas of patient care and to dedicate more time to clinical activities [56–58]. In contrast, there is no institution that regulates the professional activities of pharmacy technicians in Brazil.

Other barriers were related to the development of patient care process. Some studies have observed similar findings [32,54,59], which reinforces the need for training of pharmacists focusing on developing clinical knowledge, skills and attitudes as a strategy to promote the CPS implementation.

### Barriers related to the implementation process

One of the main barriers cited was the inappropriate period of implementation of CPS in Brazil, it is not yet known the influence of holidays on health services, unlike in the economic sector [60]. Nevertheless, pharmacists' statements and administrative reporting confirm that there is a decrease of the activities in health units, and a decrease in the demand for CPS during times of holidays and vacations.

The short period to implement CPS was also considered a barrier. The literature shows that a variability in the time needed for implementation of CPS may take 2–4 years [18,61]. This variation in the literature is explained by the multifactorial nature of the implementation process [17,18]. In other words, different participants, scenarios and types of CPS may present different barriers, facilitators, and periods required to implement the CPS.

Another barrier mentioned was the lack of an electronic health record (EHR). Several studies report the beneficial impact of EHRs on patient safety and efficiency, such as improvement in quality of care, prescribing safety, disease management, clinical documentation, work practice, preventive care, and in the volume of communication between pharmacists and the healthcare team [62–64]. There is the Brazilian National Electronic Health Record (e-SUS AB), a system that gathers all of the patient's health information and shares it with all members of the healthcare team. However, the system is restricted to primary care, and needs improved documentation by the healthcare team, and the ability to be able to share it at all levels of care.

The accredited pharmacists emphasized that the lack of prior evaluation of pharmacists’ clinical competences was a crucial barrier related to the implementation process. Detoni et al. [65] found that evaluation of human resources and pharmacists’ characteristics is essential to identify professionals more motivated, more committed to the service, and more willing to deal with the challenges associated with providing the service. As such, prior evaluation of the pharmacists might improve the success of the CPS implementation.

Finally, pharmacists mentioned that poor marketing strategies might have hampered the implementation of CPS. The studies show that marketing strategies contribute to increased visibility and prestige of the service in the community, to sensitize patients and healthcare professionals, to recruit new patients or potential users of the services, and to build a favorable image of a growing reputation and credibility in the minds of patients and healthcare professionals [66–68]. Therefore, marketing strategies may be adopted to communicate the pharmacist's clinical role to managers, the healthcare team, and the patients; this could minimize the effects of lack of understanding and awareness.

#### Barriers related to the patients

Similar to the current study, several studies showed that a lack of understanding and awareness, and resistance among the patients, were barriers to implementation of CPS [24,30,69]. Two studies reported that the pharmacist should assume a proactive role in the pharmacist-patient relationship [70,71]. In other words, the pharmacist may use marketing strategies to help patients see the value that these services can offer. Therefore, an improved pharmacist-patient relationship may lead to improved patient satisfaction and positive impacts on patients’ health outcomes.

Interestingly, barriers related to the patient did not emerge in the interviews with the managers. It is possible that the managers might have considered the patients as passive recipients of healthcare. Studies show that there is a paternalistic approach to healthcare, where health professionals make all of the decisions with little or no input from the patient [72,73]. In contrast, Vahdat et al. [74] showed that the participation of patients is not merely for consultation, but patients must participate in decisions associated with planning, performance, and evaluation of healthcare. Hence, managers should place value on patients’ participation in healthcare, and accept their role in quality of care and patient safety [75].

### Strengths and limitations

The main strength of the study was the participation of the pharmacists and the managers that worked at varied workplaces of the health system. The professionals had different backgrounds and perspectives about the same CPS implementation process. These characteristics were essential to emerge varied barriers and to have a comprehensive understanding of the implementation process.

We can list some limitations: lack of a previous participants analysis, lack of perceived barriers of other actors involved in the CPS implementation process (patients, healthcare professionals, pharmacy staff, decision makers, policymakers), the conflict of interest of some managers and some failures of the implementation process that were perceived as barriers. These limitations may have influenced the perceived barriers of pharmacists and managers who participated in the study.

## Conclusion

This study revealed the perceptions of barriers associated with the participants involved in the implementation of CPS in some public health units in a metropolis in Northeast Brazil. The accredited pharmacists, non-accredited pharmacists and managers identified barriers related to: the local healthcare networks, the healthcare team, the pharmacists, the implementation process, and the patients. These barriers fill knowledge gaps associated with the CPS implementation. Thus, this work and future studies may contribute with pharmacists, managers, decision makers, and policy makers to plan and implement CPS.
